# Hydrazine Hydrate‐Induced Surface Modification of CdS Electron Transport Layer Enables 10.30%‐Efficient Sb_2_(S,Se)_3_ Planar Solar Cells

**DOI:** 10.1002/advs.202202356

**Published:** 2022-06-26

**Authors:** Jianmin Li, Yuqi Zhao, Chuang Li, Shaoying Wang, Xueling Chen, Junbo Gong, Xiaomin Wang, Xudong Xiao

**Affiliations:** ^1^ Key Laboratory of Artificial Micro‐ and Nano‐structures of Ministry of Education and School of Physics and Technology Wuhan University Wuhan 430072 China; ^2^ Center for Biomedical Optics and Photonics (CBOP) & College of Physics and Optoelectronics Engineering Key Laboratory of Optoelectronic Devices and Systems Shenzhen University Shenzhen 518060 China

**Keywords:** CdS thin film, hydrazine hydrate, interfaces, Sb2(S,Se)_3_ solar cells, solution treatment

## Abstract

Antimony selenosulfide (Sb_2_(S,Se)_3_), a simple alloyed compound containing earth‐abundant constituents, with a tunable bandgap and high absorption coefficient has attracted significant attention in high‐efficiency photovoltaic applications. Optimizing interfacial defects and absorber layers to a high standard is essential in improving the efficiency of Sb_2_(S,Se)_3_ solar cells. In particular, the electron transport layer (ETL) greatly affects the final device performance of the superstrate structure. In this study, a simple and effective hydrazine hydrate (N_2_H_4_) solution post‐treatment is proposed to modify CdS ETL in order to enhance Sb_2_(S,Se)_3_ solar cell efficiency. By this process, oxides and residual chlorides, caused by CdCl_2_ treated CdS under a high temperature over 400 °C in air, are appropriately removed, rendering smoother and flatter CdS ETL as well as high‐quality Sb_2_(S,Se)_3_ thin films. Furthermore, the interfacial energy band alignment and recombination loss are both improved, resulting in an as‐fabricated FTO/CdS‐N_2_H_4_/Sb_2_(S,Se)_3_/spiro‐OMeTAD/Au solar cell with a high PCE of 10.30%, placing it in the top tier of Sb‐based solar devices. This study provides a fresh perspective on interfacial optimization and promotes the future development of antimony chalcogenide‐based planar solar cells.

## Graphical abstract

In this paper, a new method termed hydrazine hydrate (N_2_H_4_) assisted solution treatment (HHST) is first introduced to improve CdS electron transport layers (ETLs) quality, which enables over 10.30% planar Sb_2_(S,Se)_3_ solar cells, achieves a smoother CdS and Sb_2_(S,Se)_3_ surface, tailors the elemental composition, optimizes the defect properties and improves interfacial quality.

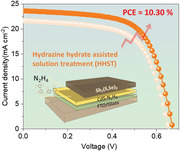

## 1 Introduction

Metal chalcogenides, including CuInGaSe_2_ (CIGS) and CdTe, have been widely used as light‐harvesting materials in solar cell technology, with a power conversion efficiency (PCE) of over 22%.^[^ ^1^, ^2^ ^]^ It is well‐known that the scarcity and toxicity of Ga and Cd elements impede their long‐term or future development. Kesterite Cu_2_ZnSn(S,Se)_4_ (CZTSSe) materials have also been considered as promising candidates for high PCE devices. However, due to its complex elemental constituents and defects, the maximum obtained PCE has remained at 12.6% for several years.^[^ ^3^ ^]^ Recently, antimony selenosulfide (Sb_2_(S,Se)_3_), an alloyed binary compound with a high absorption coefficient (>10^5^ cm^−1^), earth‐abundant non‐toxic constituents, and a tunable bandgap (through varying the atom ratio of Se and S elements), has been proposed as a potential next‐generation solar cell material.^[^ ^4^, ^5^, ^6^, ^7^, ^8^ ^]^ Meanwhile, it is encouraging that in only 6 years, the PCE based on this material at laboratory level has reached ≈10%.^[^ ^9^ ^]^ Impressively, CdS is used as the principal electron transport layer (ETL) in all Sb‐based solar cells, achieving a high efficiency of over 9%,^[^ ^4^, ^9^, ^10^, ^11^ ^]^ as well as performing well in CIGS and CdTe solar cells.^[^ ^12^ ^]^ According to a theoretical study, the CdS/Sb_2_(S,Se)_3_ interface plays an influential role in Sb‐based solar device performance, where interface recombination is the main loss mechanism, and an increase in defect density will probably result in a significant reduction in performance.^[^ ^13^ ^]^ Consequently, optimizing the CdS/Sb_2_(S,Se)_3_ interface contact while reducing interfacial defect density are the most important initiatives to improve device efficiency in the future.

In Sb‐based solar cells with a substrate structure or mature developed CIGS devices, the CdS ETL does not need to consider a CdCl_2_ post‐deposition treatment (PDT) process.^[^ ^11^, ^12^ ^]^ However, for the new Sb‐based devices with superstrate structure, treating the CdS ETL with CdCl_2_ has a proven positive effect on the superstructure CdS/Sb_2_Se_3_ solar cell.^[^ ^14^ ^]^ Moreover, the CdCl_2_ post‐treatment following chemical bath deposition (CBD)‐prepared CdS is used in most high‐efficiency Sb‐based solar devices.^[^ ^4^, ^10^, ^15^ ^]^ However, the CdCl_2_‐PDT process is executed by exposing CdCl_2_ treated CdS film to temperatures of over 400 °C in air. Cadmium chloride oxides form easily on the CdS thin film surface and have been proved to increase resistance while reducing corresponding device performance.^[^ ^16^ ^]^ Unfortunately, as Sb‐based solar cells are new, attempts to address the above problem are limited. Furthermore, exactly how cadmium chloride oxides affect CdS ETL as well as corresponding device performance is still not clear.

In this study, an effective hydrazine hydrate (N_2_H_4_) assisted solution treatment (HHST) is applied to CdS ETLs to improve the performance of Sb_2_(S,Se)_3_ thin‐film solar cells. Specifically, CdS thin film treated with CdCl_2_ solution is designated as the control sample. The control CdS is then further modified by immersing it in hydrazine hydrate (N_2_H_4_) solution for a specified period (labeled W‐HHST), followed by a hydrothermal deposition of Sb_2_(S,Se)_3_ thin film. Finally, a typical superstrate solar device with an FTO/ETL/Sb_2_(S,Se)_3_/spiro‐OMeTAD/Au structure is built. The fine reducibility and high corrosive properties of N_2_H_4_ solution^[^ ^17^, ^18^ ^]^ improve the CdS surface quality and the subsequently deposited Sb_2_(S,Se)_3_ absorber makes it smoother and flatter. Moreover, it is discovered through element composition analysis, that the remaining Cd oxychlorides on the CdS thin film surface are removed during the HHST process. Consequently, the interfacial energy band alignment and recombination loss are both improved. This results in an as‐fabricated FTO/CdS‐N_2_H_4_/Sb_2_(S,Se)_3_/spiro‐OMeTAD/Au solar cell with a high PCE of 10.30%, which could be placed in the top tier of Sb‐based solar devices. This research provides a feasible alternative CdS layer post‐treatment and a new interfacial optimization perspective, demonstrating the potential of high‐efficiency antimony chalcogenides solar cells with CdS ETLs.

## 2 Results and Discussion

**Figure 1:**
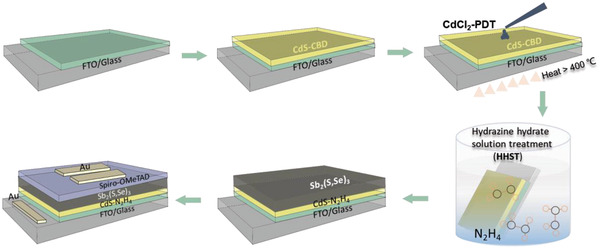
Schematic illustration of the typical fabrication process for Sb_2_(S,Se)_3_ solar cells based on CdS ETLs with HHST.

As previously reported, the CdS ETLs are fabricated using a CBD method, which is treated with CdCl_2_ solution to improve the CdS film grain size and quality. Cl and more O elements are also simultaneously incorporated into the film. It is believed that the heavy incorporation of Cl and O elements will change the basic CdS ETL properties. The interface between CdS and Sb_2_(S,Se)_3_ is also regarded as the main interface recombination for the carriers. The CdS ETL plays an important role in the final device efficiency, particularly in planar structure Sb_2_(S,Se)_3_ solar cells.^[^ ^13^ ^]^ In order to eliminate any side effect from the above elements on the interfacial properties, treating the CdS ETLs with a HHST is proposed to improve the CdS/Sb_2_(S,Se)_3_ thin‐film solar cell performance. The HHST procedure for the CdS ETLs, and Sb_2_(S,Se)_3_ solar cells with a typical structure of glass/FTO/CdS/Sb_2_(S,Se)_3_/spiro‐OMeTAD/Au, fabrication process is schematically illustrated in **Figure** **1**. Simply put, in this study, a CdS thin film deposited by a CBD method is introduced as the ETL of Sb_2_(S,Se)_3_ solar cells. CdCl_2_‐PDT is then performed on the pristine CdS thin films, in air at over 400 ℃. During the HHST process, the CdCl_2_‐treated CdS samples were immersed in hydrazine hydrate solution for various times at room temperature. The Sb_2_(S,Se)_3_ absorber layers are then synthesized by a typical hydrothermal deposition method. Finally, the planar Sb_2_(S,Se)_3_ thin‐film solar cells are assembled by spin‐coated spiro‐OMeTAD and deposition of a thermal evaporation Au layer.

**Figure 2:**
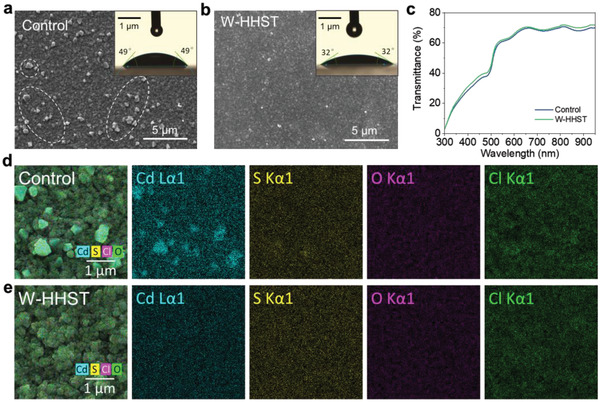
SEM images of surface morphologies for a) control CdS and b) CdS with HHST process. The contact angles of related CdS films are shown in the insets. c) Optical transmittance spectra of CdS ETLs with and without HHST. The images of surface EDS mapping for d) control‐CdS film and e) HHST processed CdS film.

As previously predicted, the HHST process influences the surface morphology and element ratios of the CdS film, changing the subsequently deposited Sb_2_(S,Se)_3_ film. Thus, to investigate the evolution of the CdS film morphology with HHST, surface characterization of the CdS film was conducted via scanning electron microscopy (SEM). As shown in **Figure** **2**a,b and Figure S1, Supporting Information, there are many small dust particles adsorbed on the CdS film surface following CdCl_2_ solution treatment. It is well‐known that a smooth and uniform surface is vital in thin‐film solar cells, used in high‐performance devices.^[^ ^19^ ^]^ Therefore, this rough CdS ETL surface may not be suitable for depositing Sb_2_(S,Se)_3_ film, and could reduce the efficiency of the final solar device. To enhance the results while considering the solubility of cadmium chlorides, Figure S2, Supporting Information, shows the SEM images of a CdS thin film treated with just water. However, several particles are still absorbed on the CdS thin films after being treated purely with water. In contrast, impressively, on the CdS film etched with HHST, the dust particles decreased dramatically and the film became smoother and flatter, compared to the pristine CdS sample. Moreover, the related CdS film contact angles are assessed and presented in the insets. The pristine CdS film exhibited a contact angle of ≈49° (Figure 2a inset), whereas the W‐HHST CdS thin films exhibited smaller contact angles of ≈32° (Figure 2b inset). This implies an increased water affinity initiated by the N_2_H_4_ post‐treatment. Increased water affinity may prove favorable in subsequent Sb_2_(S,Se)_3_ thin‐film depositions in water.

To better understand the CdS film changes and evolution of these particles, other CdS thin‐film characteristics, including optical properties, crystalline structure and elemental composition, are further investigated via UV–vis, XRD and energy dispersive spectroscopy (EDS), respectively. As shown in Figure 2c, the CdS film with HHST transmittance is slightly higher than the control sample, in the whole visible range, which is beneficial to the light transmission and absorption of the Sb_2_(S,Se)_3_ layer. The reason for higher transmittance is probably the corrosive ability of N_2_H_4_ solution to clear any particles formed on the surface, leading to a smoother CdS film surface. Grazing incidence angle X‐ray diffraction (GIXRD) tests are performed to investigate the HHST phase evolution, as shown in Figure S3, Supporting Information. It can be clearly seen that all the patterns correspond well with the CdS structure (JCPDS No. 41–1049), suggesting that the HHST process has no adverse effects on the CdS thin film crystal structure. Therefore, it is reasonable that the CdS HHST process shows a negligible influence on the Sb_2_(S,Se)_3_ thin‐film crystallization (Figure S4, Supporting Information). To intuitively observe the change in the particles’ element composition, EDS mappings are characterized. As shown in Figure 2d,e, it is confirmed that the particles on the control CdS film surface are rich in Cd and Cl. When the CdS film is treated with HHST, the content of Cd and Cl is reduced. However, the O element change is relatively indistinct, which is predicted due to the SnO_2_ signal disturbance in the FTO substrate. Thus, a surface‐sensitive analysis using X‐ray photoemission spectroscopy (XPS) is conducted to further identify the effect of HHST on element composition of the CdS surface.

**Figure 3:**
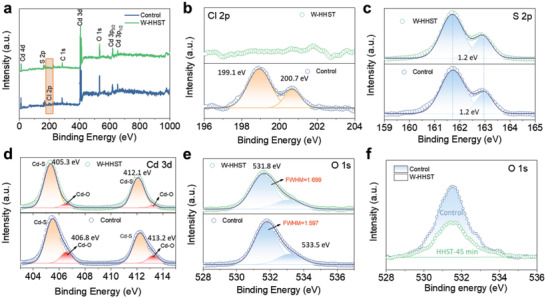
a) The full XPS spectra of CdS films with control and HHST. b–e) XPS peaks of Cl 2p, S 2p, Cd 3d, and O 1s for CdS (control) and HHST processed CdS film. Gaussian fitting was applied to analyze the data. f) XPS peaks of O 1s for a different time for HHST.

The XPS full survey spectra and fine spectra data of Cl 2p, S 2p, Cd 3p, and O 1s for the control and HHST processed CdS films are shown in **Figure** **3**, and the binding energy (BE) is calibrated by referencing the C1s signal of 285.3 eV. The Cl 2p XPS spectrum of the control CdS film shows two obvious peaks, at binding energies of 199.1 and 200.7 eV, respectively, which are well corresponding to the reported BE of Cl 2p.^[^ ^20^ ^]^ However, no Cl 2p peaks are detected in the HHST‐treated CdS film spectrum. This indicates that the residual surface Cd oxychloride caused by CdCl_2_‐PDT, is decreased by the HHST process, which is consistent with the EDS results. In terms of the S 2p XPS spectra shown in Figure 3c, the peaks of each sample exhibit no obvious change in BE, while showing a 1.2 eV difference. This is consistent with the divalent state of sulfur in CBD CdS.^[^ ^21^, ^22^ ^]^ While for the Cd 3d XPS spectra in Figure 3d, the main peaks at 405.3 and 412.1 eV are attributed to Cd—S in CdS,^[^ ^23^, ^24^ ^]^ and the extra peaks at 406.8 and 413.2 eV originate from the Cd—O in CdS.^[^ ^25^ ^]^ This demonstrates a significant decrease in the Cd—O peak following HHST. It was predicted that the N_2_H_4_ solution etched the control CdS film, causing the intensity difference on Cd 3d XPS spectra. This phenomenon is more obvious when comparing the XPS spectra of O 1s. In Figure 3e, the peak at 531.8 eV indicates the presence of Cd—O^[^ ^14^, ^26^, ^27^ ^]^ and the peak at 533.5 eV is geared to C—O.^[^ ^26^ ^]^ Cd—O from the cadmium oxide is expected during the high‐temperature annealing for CdS film, and the C—O mainly contributes to the CdS film surface pollution, causing the O 1s peak in both samples. On comparing the peaks at 531.8 eV, we find that the Cd—O peak intensity dropped slightly and has a larger full width at half maximum (FWHM) for the treated CdS sample, reflecting the reduction of Cd oxychloride content by the HHST. Moreover, to enlarge the etching effect, we also measure the evolution of the O 1s peak of the CdS‐HHST film over a long time (≈45 min). It clearly demonstrates that the Cd—O peak intensity becomes weaker with increased HHST time for the CdS films, as presented in Figure 3f. As detailed above, the HHST process described here may successfully adjust the CdS film surface properties, due to its fine reducibility and high corrosive ability. This results in CdS ETLs with greater transmittance, a smoother surface appearance, and less Cd oxychlorides.

**Figure 4:**
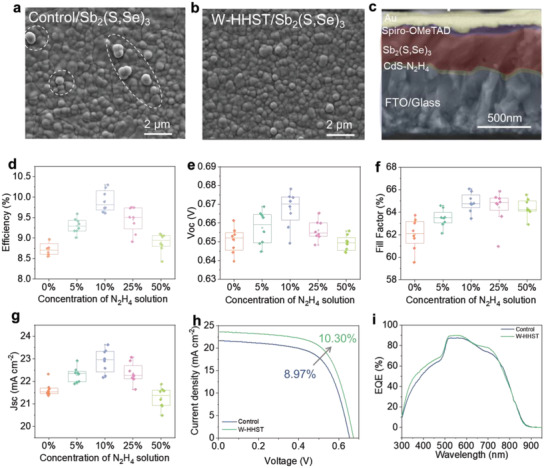
a,b) SEM images of Sb_2_(S,Se)_3_ films deposited on pristine CdS layer and on CdS with HHST, respectively. c) A cross‐sectional SEM image of Sb_2_(S,Se)_3_ solar cell. d–g) Parameters evolution of Sb_2_(S,Se)_3_ devices with CdS ETLs treated with different concentrations of N_2_H_4_ solution. h) The *J*–*V* curves and i) EQE spectra of champion Sb_2_(S,Se)_3_ solar cells based on control and HHST processed CdS ETLs.

The surface SEM images of the subsequently deposited Sb_2_(S,Se)_3_ absorbed layers by hydrothermal deposition method on CdS films with or without HHST have also been compared in **Figure** **4**a,b. Interestingly, this shows that the Sb_2_(S,Se)_3_ film also becomes much flatter than the control sample, due to fewer dust particles and higher water affinity in CdS with HHST, which is more favorable for the adsorption and growth of Sb_2_(S,Se)_3_. According to the literature surveys, during a typical hydrothermal deposition process, the Sb_2_(S,Se)_3_ homogeneous and heterogeneous nucleation rates decrease exponentially with homogeneous Gibbs free energy (Δ*G*_Hom_) and heterogeneous Gibbs free energy (Δ*G*_Het_), respectively. The homogeneous and the heterogeneous Gibbs free energy can be expressed by the following equations:^[^ ^28^, ^29^ ^]^

(1) $$\Delta G_{\mathrm{Hom}}=\frac{4}{3}\;\pi r_{k}^{3}n\Delta G_{v}+4\pi r_{k}^{2}n\gamma_{\mathrm{SL}}$$

(2) $$\Delta G_{\mathrm{Het}}=\Delta G_{\mathrm{Hom}}f\left(\theta \right),\;0<f\left(\theta \right)<1$$

where *r*_k_ is the critical radius of the nuclei, *n* is the number of critical nuclei per unit volume, Δ*G*_v_ is the change in free energy between the nuclei and solution per unit volume, *γ*_SL_ is the particle surface energy, and *θ* is the contact angle. Theoretically, according to the Equation (2), a smaller contact angle results in lower Gibbs free energy for heterogeneous nucleation, thereby increasing the nucleating density and promoting the film densification process. Conversely, some Sb_2_(S,Se)_3_ particles are formed on the surface of the Sb_2_(S,Se)_3_ layer for untreated CdS film, which could adversely affect the spin‐coating process for the spiro‐OMeTAD layer and the final Sb_2_(S,Se)_3_ solar cell efficiency.

Although high‐quality CdS ETLs and Sb_2_(S,Se)_3_ absorber layers are obtained through the HHST approach, its effects on the photovoltaic properties of Sb_2_(S,Se)_3_ solar cells is unclear. Two types of CdS ETL‐based Sb_2_(S,Se)_3_ solar cells with an FTO/CdS/Sb_2_(S,Se)_3_/spiro‐OMeTAD/Au planar structure. Figure 4c intuitively shows a cross‐sectional SEM image of a typical solar cell device based on HHST processed CdS ETLs. The corresponding thickness of CdS, Sb_2_(S,Se)_3_ and spiro‐OMeTAD layers are ≈50, ≈280, and ≈80 nm, respectively. The HHST process parameters are carefully and systematically studied, including the N_2_H_4_ solution concentration and HHST duration time on the CdS thin films. The current density–voltage (*J*–*V*) characteristics of the Sb_2_(S,Se)_3_ solar cells are investigated under one sun illumination (AM 1.5G). In Figure 4d–g and Figure S5, Supporting Information, the results demonstrate that CdS film treated with the 10% concentration N_2_H_4_ solution at room temperature for only 4 min delivers the best device performance, which is attributed to the significantly improved current density (*J*_sc_) and fill factor (FF) value due to the increased transmission and reduced impurities of the CdS film. In this case, we also optimize the deposition temperature (Figure S6, Supporting Information), growth time (Figure S7, Supporting Information), and annealing temperature (Figure S8, Supporting Information) of Sb_2_(S,Se)_3_ film based on the CdS layer with the best HHST process parameters. Finally, Sb_2_(S,Se)_3_ solar cells based on the control and HHST‐CdS ETLs exhibit PCEs of 8.97% and 10.30%, respectively, corresponding *J*–*V* curves are presented in Figure 4h. The enhancement in PCE for HHST‐CdS‐based devices can mainly be attributed to the increased *J*_SC_ values, from 22.33 to 23.63 mA cm^−2^, and fill factor (FF) values, from 63.74% to 66.07%. The detailed photovoltaic parameters of each device are displayed in **Table** **1**. To explore other differences between the control‐CdS and HHST‐CdS based Sb_2_(S,Se)_3_ devices, external quantum efficiency (EQE) measurements are taken and the results are displayed in Figure 4i. As expected, the HHST‐CdS based Sb_2_(S,Se)_3_ device spectrum increases in short wavelengths below 500 nm, which can be explained by the increase in HHST‐treated CdS film optical transmittance. More importantly, the spectrum of Sb_2_(S,Se)_3_ device with HHST also shows a slight increase between 600 and 750 nm. This may be due to the improvement of interfacial property between HHST optimized CdS and Sb_2_(S,Se)_3_ film. Furthermore, as illustrated in Figure S9, Supporting Information, numerous pinholes are generated on the CdS ETL surface following high concentration N_2_H_4_ etching. Therefore, the breaking of the CdS ETL surface is proposed as one explanation for the decreased Sb_2_(S,Se)_3_ solar cell performance with increasing N_2_H_4_ concentrations.

**Table 1:** Champion and average device parameters for solar cells based on different N_2_H_4_ concentrations

Sample	*V*_oc_ [V]	PCE [%]	FF [%]	*J*_sc_ [mA cm^−2^]
Control	0.651 ± 0.011 (0.661)	8.73 ± 0.24 (8.97)	62.04 ± 1.69 (63.74)	21.63 ± 0.69 (22.33)
5%	0.657 ± 0.011 (0.669)	9.30 ± 0.29 (9.59)	63.47 ± 1.13 (64.59)	22.29 ± 0.63 (22.92)
10%	0.667 ± 0.011 (0.678)	9.90 ± 0.40 (10.30)	64.86 ± 1.21 (66.07)	22.90 ± 0.73 (23.63)
25%	0.656 ± 0.009 (0.665)	9.45 ± 0.30 (9.75)	64.42 ± 1.44 (65.85)	22.36 ± 0.71 (23.07)
50%	0.649 ± 0.006 (0.656)	8.89 ± 0.21 (9.10)	64.39 ± 1.16 (65.55)	21.27 ± 0.61 (21.88)

**Figure 5:**
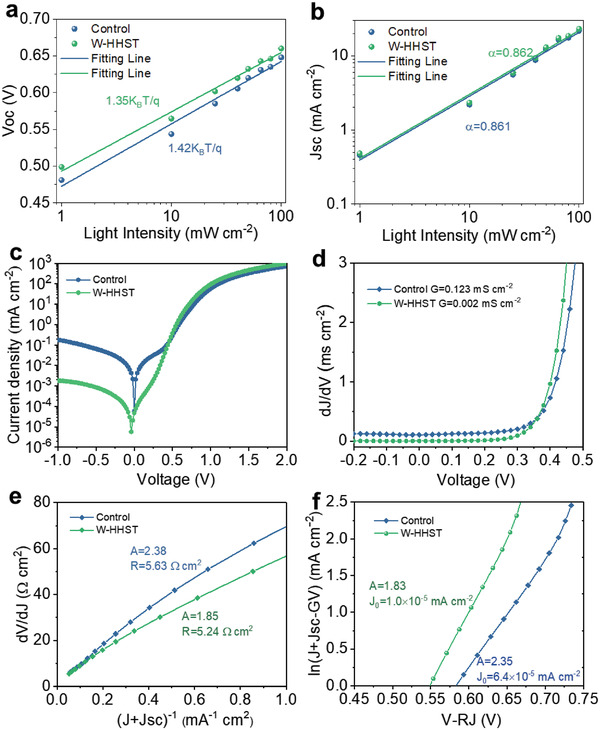
a,b) The plots of *V*_oc_ and *J*_sc_ versus log‐scaled light intensity of solar cells based on CdS with or without HHST. c–f) Dark *J*–*V* characteristics of Sb_2_(S,Se)_3_ solar cells based on control CdS and HHST processed CdS. c) Dark *J*–*V* curves, d) characterization of shunt conduction G, e) the plots of d*V*/d*J* versus (*J* + *J*_SC_), and f) the curves of ln(*J* + *J*_SC_ − *GV*) against (*V* − *RJ*) to determine A, *R*_S_, and *J*_0_.

To gain more insight into the carrier transport and recombination information, the relationship between light intensity (*I*), *V*_OC_, and *J*_SC_ is investigated (Figure S10, Supporting Information). The dependence of *V*_OC_ on *I* can be expressed by *V*_OC_ = Δ*kT*ln (*I*)/*q*. The *V*_OC_ versus log‐scaled intensity is shown in **Figure** **5**a, in which *V*_OC_ increases linearly with the logarithm of light intensity. By fitting, each device shows *ɛ* values of 1.42 (Control) and 1.35 (W‐HHST), respectively. A lower *ɛ* value for the W‐HHST device indicates that there is less trap‐assisted Shockley–Read–Hall recombination in Sb_2_(S,Se)_3_ solar cells.^[^ ^30^, ^31^ ^]^ Besides, the dependence of *J*_SC_ on *I* can be defined as *J*_SC_∝ *I* ^*α*^ .^[^ ^32^ ^]^ As usual, the *α* value is less than 1,^[^ ^33^ ^]^ and higher *α* values suggest a better charge collection in the device.^[^ ^34^ ^]^ Here, the relationship of *J*_SC_ with *I* is shown in Figure 5b. Two devices deliver similar *α* values of 0.862 and 0.861,^[^ ^4^, ^35^ ^]^ which are consistent with previously reported values of high‐efficiency Sb_2_(S,Se)_3_ solar cells.

To clearly understand the reduced recombination loss in W‐HHST based Sb_2_(S,Se)_3_ solar cells, *J*–*V* characteristics under dark for each device are also measured and analyzed. Figure 5c displays the dark *J*–*V* plots for two devices based on the control and HHST‐CdS ETLs. By plots fitting, the shun conduction (*G*), diode ideality factor (*A*), series resistance (*R*), and reverse saturation current density (*J*_0_) can be calculated according to the following Equation (3):

(3) $$J=J_{0}\exp\left[\frac{q}{AkT}\left(V-RJ\right)\right]+GV-J_{L}$$

where k is the Boltzmann constant, *T* is the temperature under actual conditions, and *q* represents the quantity of electric charge. The plots of d*J*/d*V* against *V* for two devices are presented in Figure 5d, where the *G* values are obtained by reading from the flat value under reverse bias. The values of the two devices without and with HHST are 0.123 and 0.002 mS cm^−2^, respectively, indicating a higher shun resistance for the device with HHST‐CdS ETL. Besides, for the dark *J*–*V* tests, the *J*_SC_ is set as zero. By linear fitting the plots of d*V*/d*J* versus (*J* + *J*_SC_), the *A* value can be calculated by the slope of *A*k*T*/*q*, and *R* can be obtained from the intercept. As illustrated in Figure 5e, the values of *A* and *R* are determined as 2.38 and 5.63 Ω cm^2^ for the control device, and 1.85 and 5.24 Ω cm^2^ for the device by employing HHST treated CdS, respectively. Moreover, *A* and *J*_0_ values can be extracted from fitting the curves of ln(*J* + *J*_SC_ − *GV*) versus (*V* − *RJ*), as presented in Figure 5f. The *A* values for each device are similar to those obtained from Figure 5e, and the *J*_0_ decreases from 6.4 × 10^−5^ to 1.0 × 10^−5^ mA cm^−2^ due to the effective action of HHST on the CdS ETL. In summary, the HHST action on the CdS ETL results in smaller *J*_0_ and *A*, and larger shun resistance, which induces a lower recombination probability and less carrier loss. Therefore, HHST‐optimized CdS ETLs with a smooth surface and less oxychloride probably lead to an improved CdS/Sb_2_(S,Se)_3_ heterojunction, thus yielding an enhancement in *J*_SC_ and FF.

**Figure 6:**
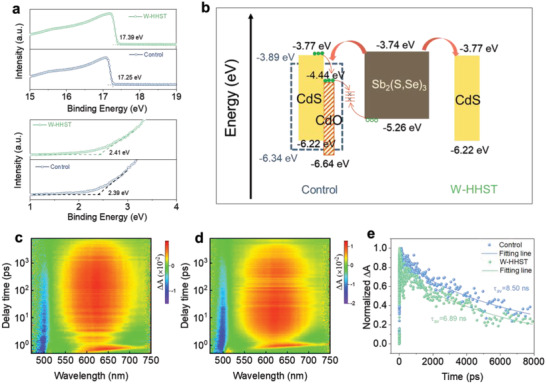
a) Second electron cutoff region and valence band position of CdS films for different treatments by UPS measurements. b) Energy band alignment diagram for Sb_2_(S,Se)_3_ solar cell devices. c,d) TAS mappings of Sb_2_(S,Se)_3_ films deposited on pristine CdS and HHST‐CdS. e) Transient decay kinetics (scatter) and curve fitting (solid lines) monitored at 632 nm wavelength for Sb_2_(S,Se)_3_ films deposited on CdS layers under control and with HHST.

In order to explain the underlying mechanism behind the FF and *J*sc enhancement in W‐HHST based Sb_2_(S,Se)_3_ solar cells, ultraviolet photoelectron spectroscopy (UPS) measurement was used to investigate the interfacial charge transfer. As a result, the Fermi level of untreated CdS film is calculated to be −3.95 eV, and the valence band is ≈2.39 eV under the Fermi level (**Figure** **6**a). Consequently, the conduction band is about −3.89 eV, according to the bandgap (2.45 eV) obtained from the optical transmission spectra in Figure 1c. In comparison, the Fermi level of CdS ETLs based on HHST is upshifted to −3.81 eV and the valence band is ≈2.41 eV under the Fermi level, thus the conduction band can be calculated as −3.77 eV (Figure 6a). According to relevant literature, the positions of the conduction band and valence band for CdO are determined as −4.44 and −6.64 eV, and those of Sb_2_(S,Se)_3_ are determined as −3.74 and −5.26 eV, respectively.^[^ ^36^, ^37^ ^]^ Thus, the upshift of Fermi level for CdS sample with HHST is owing to the reduction of CdO in the CdS film, resulting from the reducibility and corrosivity of hydrazine hydrate solution. Finally, the band alignment of CdS ETLs with the Sb_2_(S,Se)_3_ absorber is illustrated in Figure 6b. As expected, in CdS film not treated with HHST, it is more likely that the electrons and holes recombine at the interface due to the lower conduction band position of CdO, consequently yielding more carrier recombination loss as well as deteriorated performance for final solar devices. Furthermore, as shown in Figures S11 and S12, Supporting Information, there are no obvious improvements in the performance of Sb_2_(S,Se)_3_ solar cells based on CdS ETLs treated just with water, indicating that cadmium chlorides solubleness in water is irrelevant. Therefore, the values of FF and *J*_sc_ are significantly improved by etching the impurities on CdS surface with HHST, making interface contact between the CdS and Sb_2_(S,Se)_3_ layer to be more matching in the present solar cell.

To further explore the effect of the HHST process on the carrier transport dynamics, we performed transient absorption spectroscopy (TAS) characterizations. Here, the time window of time‐resolved transient spectra is 0–8000 ps. A 400 nm pulse laser was used to illuminate the Sb_2_(S,Se)_3_ films. Additionally, we selected CdS/Sb_2_(S,Se)_3_ and HHST‐CdS/Sb_2_(S,Se)_3_ films as samples for the comparison, which directly reflect that the electron extraction efficiency of the CdS ETL. According to the TAS mappings in Figure 6c,d, two absorption peaks near 450 and 650 nm are detected in two types of films, which can be ascribed to the characteristic absorption of the CdS and Sb_2_(S,Se)_3_ films, according to the previously reported TA evolution of the CdS/Sb_2_(S,Se)_3_ sample. We then extracted the decay kinetics of two films at the wavelength of 632 nm (Figure 6c). The kinetics curve is fitted by a biexponential decay model. After fitting, the carrier lifetime for the CdS/Sb_2_(S,Se)_3_ and HHST‐CdS/Sb_2_(S,Se)_3_ films are 8.50 and 6.89 ns (Table S1, Supporting Information), respectively. The decreased carrier lifetime of HHST‐CdS/Sb_2_(S,Se)_3_ exactly indicates that the electrons transferred from the Sb_2_(S,Se)_3_ absorber to the HHST‐CdS ETL is faster than in the control sample, resulting in less recombination with holes. Hence, the TAS study further explains the lower recombination probability and improved device performance of the HHST‐CdS/Sb_2_(S,Se)_3_ device, consistent with previous electrical property characterizations.

## 3 Conclusions

In summary, we propose an effective HHST for CdS films and reveal its positive effects on the properties of CdS ETLs as well as the performance improvement of Sb_2_(S,Se)_3_ thin‐film solar cells. It conclusively points out that using the HHST technique improves the device performance in three aspects: i) the obliteration of residual Cd oxychloride by HHST decreases the number of dust particles on the CdS surface, creating a smoother surface, higher optical transparency of CdS film and an obvious increment in the EQE of the final solar device; ii) CdS with HHST facilitates the deposition of Sb_2_(S,Se)_3_ thin films with high quality, which benefits carrier collection; and iii) HHST improves the energy band alignment and increase the high electron transportation efficiency between CdS and Sb_2_(S,Se)_3_ interface, indicating a positive effect on the reduction in the carrier recombination loss. Finally, by carefully optimizing the relevant fabrication parameters, a PCE of 10.30% was achieved for HHST‐based Sb_2_(S,Se)_3_ solar cell. This study offers an effective approach for the surface passivation of CdS ETLs, which benefits the future application of high‐performance chalcogenide thin‐film solar cells.

## 4 Experimental Section

### Preparation of the CdS ETL

The CdS film was deposited on an F‐doped SnO_2_ (FTO) substrate using a traditional CBD method. The FTO substrates were cleaned sequentially with deionized water, acetone and ethanol, respectively. During the CdS film CBD process, a precursor solution was prepared by mixing 20 mL Cd(NO_3_)_2_ (1.5 mm), 13 mL thiourea (1.5 m), 26 mL ammonia (25–28%), and 140 mL deionized water. The deposition process was set at 63 °C for 16 min with the substrate placed face down in the growth solution. After growth, the CdS film CdCl_2_‐PDT was executed by spin‐coating a CdCl_2_·2.5H_2_O methanol solution with a 20 mg mL^−1^ concentration at 3000 rpm for 30 s, followed by annealing at 430 °C for 15 min, in air.

### Hydrazine Hydrate Assisted Solution Treatment (HHST) of CdCl_2_‐Treated CdS Thin Film

When the CdCl_2_‐treated CdS film had cooled to room temperature naturally, the samples were immersed in hydrazine hydrate solution for different durations at room temperature. The relevant experimental parameters were then optimized, including the N_2_H_4_ solution concentration and treatment time. It should be noted that a 50% mass fraction of hydrazine hydrate solution diluted at a 5 % volume ratio is simply referred to as 5%. After being removed from the N_2_H_4_ solution, the samples were rinsed with deionized water. Finally, the as‐fabricated FTO/CdS‐N_2_H_4_ samples were blow‐dried using nitrogen gas.

### Fabrication of Sb_2_(S,Se)_3_ Thin Films and Solar Devices

Sb_2_(S,Se)_3_ films were synthesized utilizing a typical hydrothermal method with precursor materials including KSbC_4_H_4_O_7_·0.5H_2_O, Na_2_S_2_O_3_·5H_2_O, CH_4_N_2_Se, and deionized water. In brief, 4 mmol KSbC_4_H_4_O_7_·0.5H_2_O and 16 mmol Na_2_S_2_O_3_·5H_2_O were mixed and stirred in 200 mL deionized water. 0.8 mmol CH_4_N_2_Se was then added and the mixture was stirred until the solution clarified. It was then transferred into autoclave Teflon tanks containing samples with two different CdS thin film types. The autoclaves were placed in an air oven for 110 min at 120 °C to deposit the Sb_2_(S,Se)_3_ film. Subsequently, the as‐grown Sb_2_(S,Se)_3_ films were annealed at 350 °C for 10 min in a nitrogen atmosphere. For the device fabrication, 2,2′,7,7′‐tetrakis(N,N‐di[4‐methoxyphenyl] 39 amino)‐9,9′‐spirobifluorene (spiro‐OMeTAD), fabricated according to previous reports,^[^ ^4^, ^10^, ^36^ ^]^ was selected as the hole transport layer. Typical superstrate Sb_2_(S,Se)_3_ planar solar cells with the structure of glass/FTO/ETLs/Sb_2_(S,Se)_3_/spiro‐OMeTAD/Au, were constructed after thermal evaporation using gold electrodes. Finally, a mask with an aperture area of 0.0735 cm^[^ ^2^ ^]^ was adopted to define the active device area.

### Characterizations

The surface and sectional morphologies of CdS and Sb_2_(S,Se)_3_ films were characterized using a SEM (FEI, Sirion 200), equipped with EDS. The element composition and chemical states of the CdS films were analyzed via X‐ray photoelectron spectroscopy (XPS) (ThermoFisher, ESCALAB 250Xi). The crystallinity and phase of the CdS samples were detected using grazing incidence angle X‐ray diffraction (GIXRD). The *J*–*V* curves of the Sb_2_(S,Se)_3_ devices were measured under AM 1.5G, 100 mW cm^−2^ conditions, with illumination provided by a solar simulator (Enlitech, SS‐X100R), and were recorded using a source meter (Keithley Instruments, 2400). The EQE was investigated using a quantum efficiency (QE) measurement system (Enlitech, QE‐R666). The energy band positions of two types of CdS thin film were determined via ultraviolet photoelectron spectroscopy (UPS) (Thermo Fisher, ESCALAB 250Xi) with a bias of −5 V. The optical transmittance of the samples was detected by ultraviolet‐visible near‐infrared absorption spectrum (UV–vis–NIR, Agilent Cary 5000). The ultrafast transient absorption (TA) of the CdS/Sb_2_(S,Se)_3_ films was performed on a pump‐probe system (Helios, Ultrafast System) with a maximum time delay of ≈8 ns using a motorized optical delay line under ambient conditions. The pump pulses at 400 nm (≈200 µW average power at the sample) were delivered by an ultrafast optical parametric amplifier (OPera Solo) excited by a regenerative amplifier (Coherent Astrella, 800 nm, 35 fs, 5 mJ, 1 kHz), seeded with a mode‐locked Ti:sapphire oscillator (Coherent Vitara, 800 nm, 80 MHz), and pumped with an LBO laser (Coherent Evolution‐50C, 1 kHz system). A small number of 800 nm femtosecond pulses from the regenerative amplifier were used to pump a sapphire crystal, which created a 420–780 nm white light continuum as the probe pulses. The data was fitted by a multi‐exponential function:

(4) $$\Delta A\left(t\right)=\sum_{i=1}^{N}A_{i}\exp\left(-t/\tau_{i}\right)$$

where *t* is the probe time delay, and *A*_i_ and *τ*_i_ are the amplitude and decay lifetime. The number of components *N* to satisfactorily fit the experimental data is two. The average lifetime *τ*_ave_ was estimated from the fitting parameters according to the following equation *τ*_ave_ = Σ*A*_i_ *τ*_i_^2^/Σ*A*_i_ *τ*_i_.

## Conflict of Interest

The authors declare no conflict of interest.

## Supporting information

Supporting information

## Data Availability

The data that support the findings of this study are available in the supplementary material of this article.
