# Supplementary material for: Hydrazine Hydrate‐Induced Surface Modification of CdS Electron Transport Layer Enables 10.30%‐Efficient Sb2(S,Se)3 Planar Solar Cells
Source: Adv Sci (Weinh). 2022 Jun 26;9(25):2202356. doi: 10.1002/advs.202202356 (PMC9443468; doi:10.1002/advs.202202356)
Supplement: Supplementary file 1 — Supporting information [file ADVS-9-2202356-s001.pdf]

ADVANCED SCIENCE
Open Access

Supporting Information

for *Adv. Sci.*, DOI 10.1002/advs.202202356

Hydrazine Hydrate-Induced Surface Modification of CdS Electron Transport Layer Enables 10.30%-Efficient $Sb_2(S,Se)_3$ Planar Solar Cells

Jianmin Li, Yuqi Zhao, Chuang Li, Shaoying Wang, Xueling Chen, Junbo Gong, Xiaomin Wang* and Xudong Xiao**

Supporting Information

Hydrazine hydrate-induced surface modification of CdS electron transport layer enables 10.30%-efficient $Sb_2(S,Se)_3$ planar solar cells

Jianmin Li[a]*, Yuqi Zhao[a], Chuang Li[a], Shaoying Wang[a], Xueling Chen[a], Junbo Gong[a], Xiaomin Wang[b]*, and Xudong Xiao[a]*

a. Key Laboratory of Artificial Micro- and Nano-structures of Ministry of Education, and School of Physics and Technology, Wuhan University, Wuhan 430072, China.

b. Center for Biomedical Optics and Photonics (CBOP) & College of Physics and Optoelectronics Engineering, Key Laboratory of Optoelectronic Devices and Systems, Shenzhen University, Shenzhen, 518060, P. R. China

E-mail: ljmphy@whu.edu.cn, cathy1@mail.ustc.edu.cn, xdxiao@whu.edu.cn

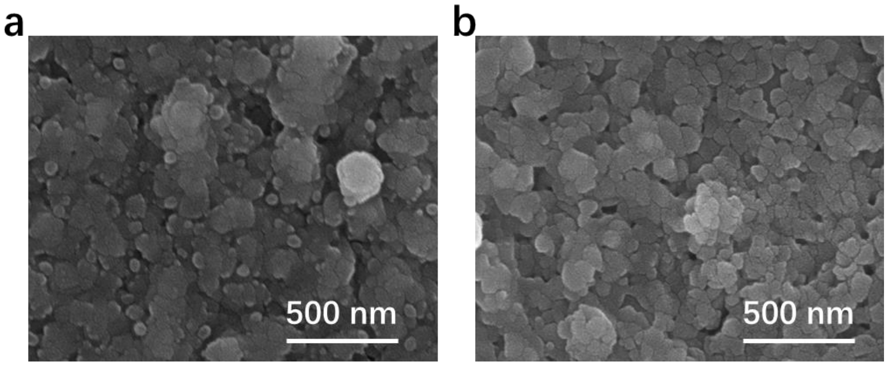

Figure S1 SEM images with higher magnification of surface morphologies for (a) control CdS and (b) CdS with HHST process.

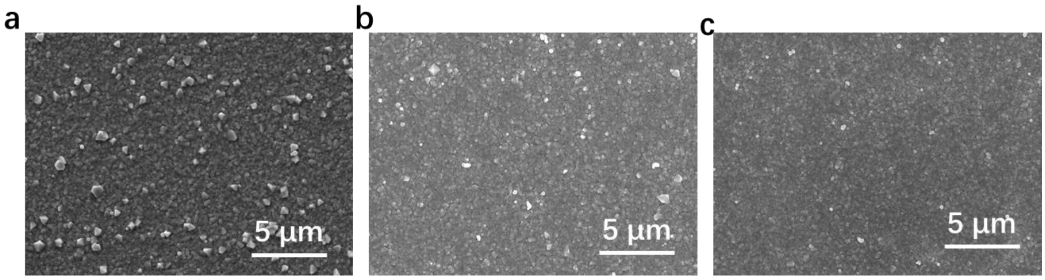

Figure S2 SEM images of surface morphologies for (a) control CdS, (b) CdS with water, and (c) CdS with HHST process.

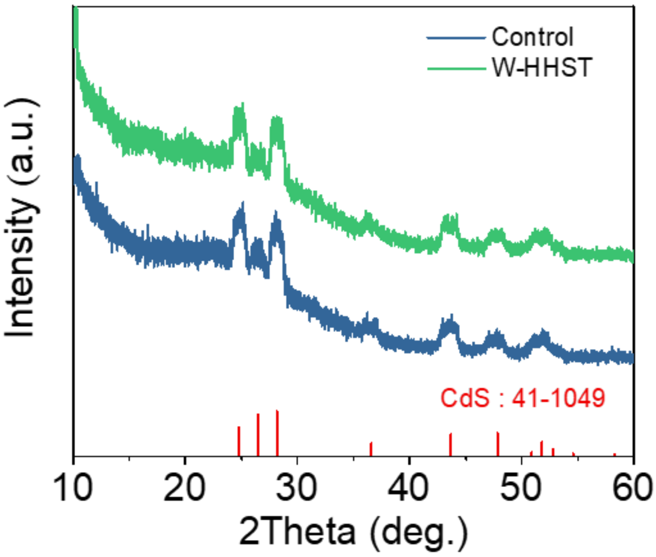

Figure S3 GIXRD spectra of control CdS films or without HHST.

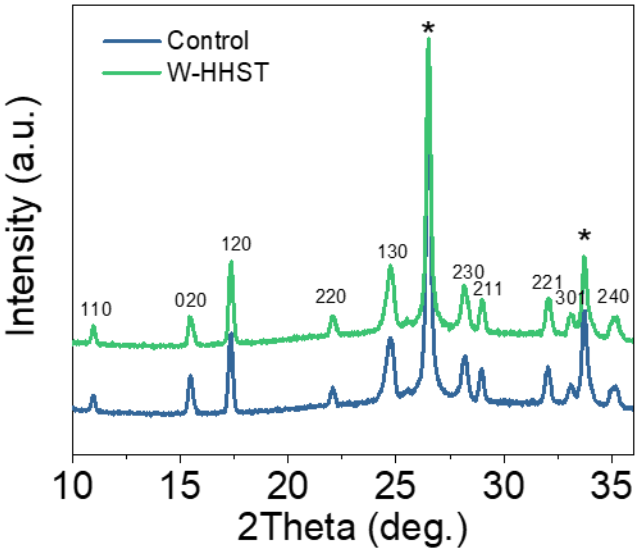

Figure S4 XRD spectra for $Sb_2(S,Se)_3$ thin films deposited on CdS layers with or without HHST. The peaks from FTO are denoted by (*).

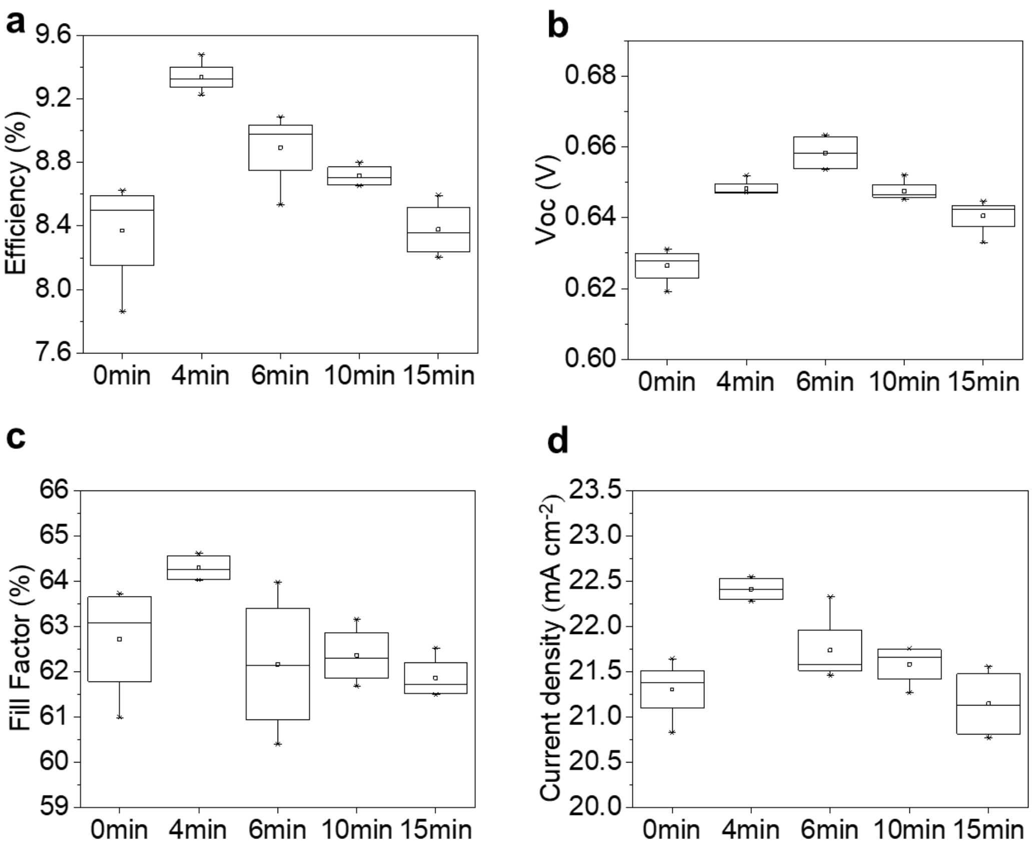

Figure S5 Statistical boxplots of efficiency (%), Voc (V), fill factor (%), and current density (mA cm^{-2}) for 10% hydrazine hydrate with different immersed times (0 min-15 min).

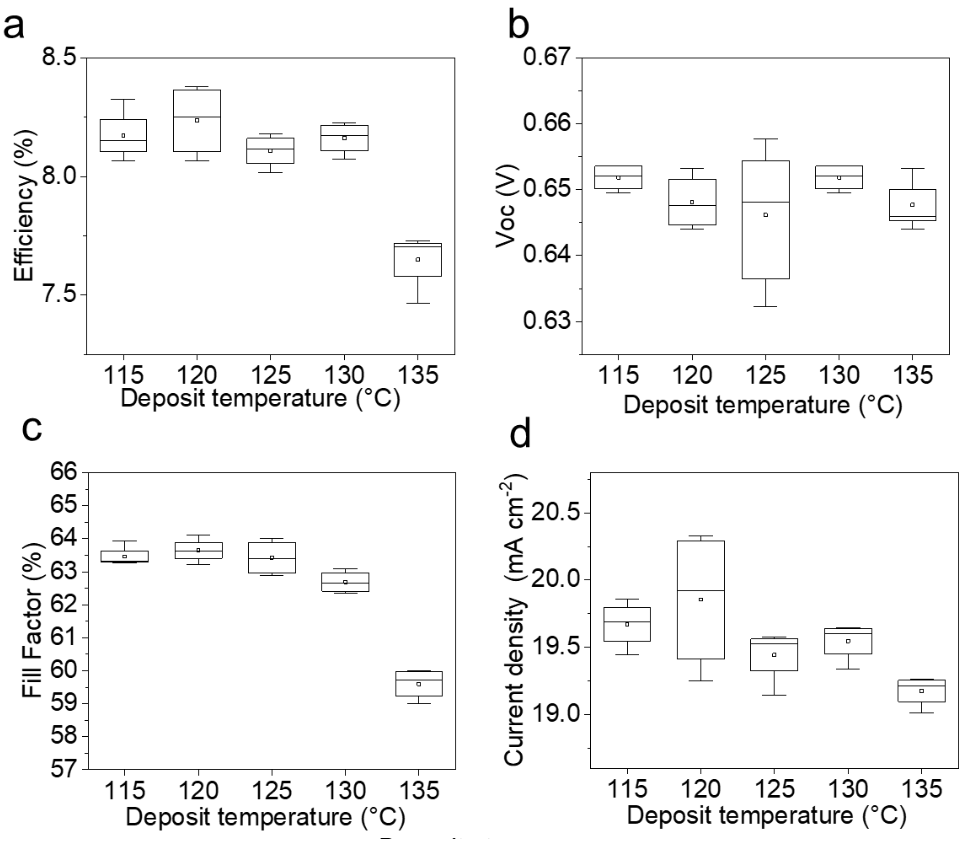

Figure S6 (a-d) Statistical boxplots of efficiency (%), Voc (V), fill factor (%), and current density (mA cm^{-2}) for different growth temperatures of $Sb_2(S,Se)_3$ thin films.

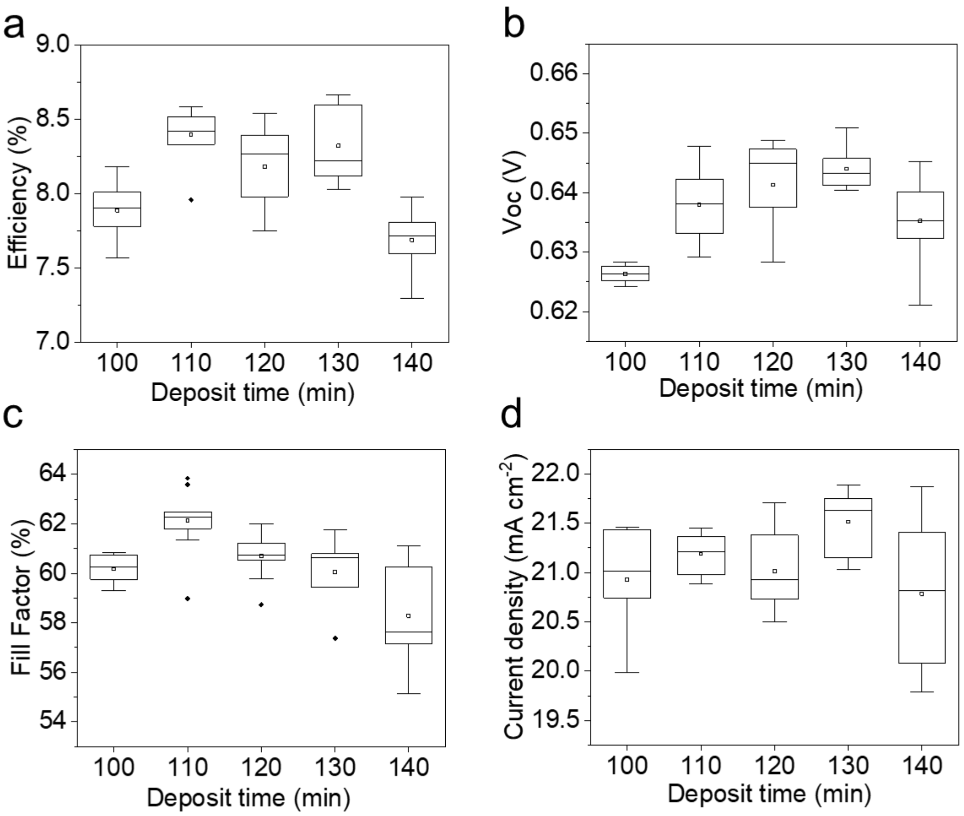

Figure S7 (a-d) Statistical boxplots of efficiency (%), Voc (V), fill factor (%), and current density (mA cm^{-2}) for different growth times of $Sb_2(S,Se)_3$ thin films at 120°C.

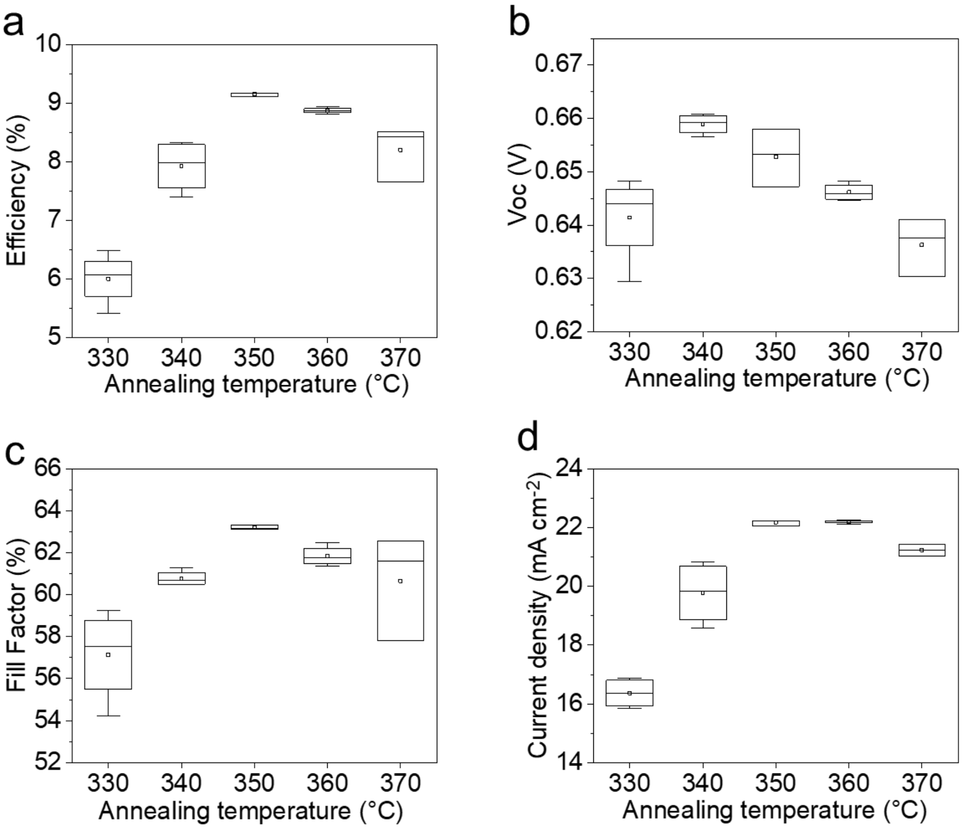

Figure S8 (a-d) Statistical boxplots of efficiency (%), Voc (V), fill factor (%), and current density (mA cm^{-2}) for different annealing temperatures of $Sb_2(S,Se)_3$ thin films.

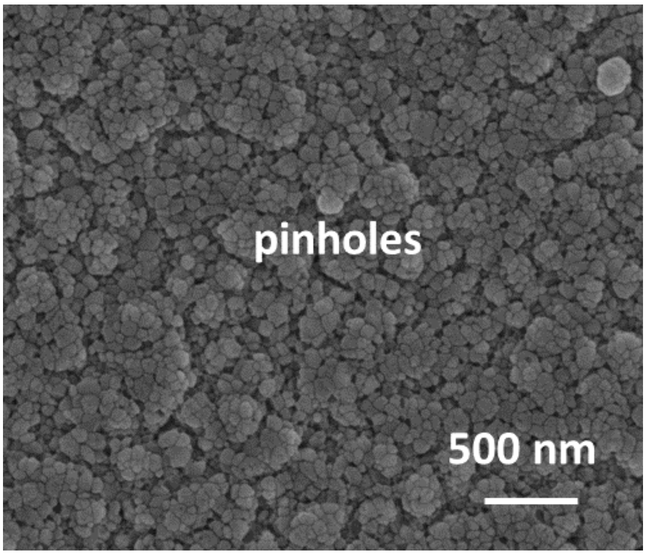

Figure S9 SEM image of CdS thin film etched with high concentration (25%) of N_2H_4 for 4min.

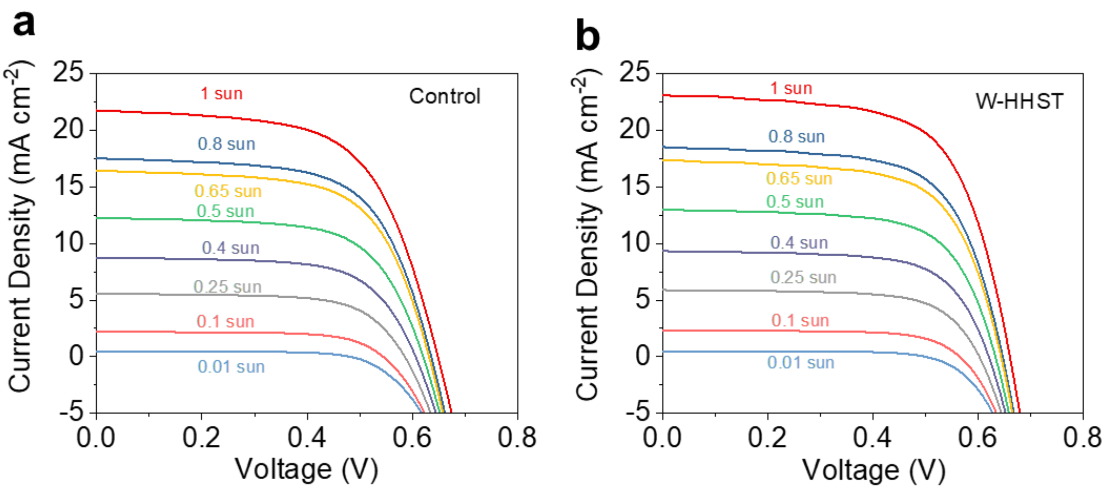

Figure S10 Light intensity-dependent J-V curves of $Sb_2(S,Se)_3$ solar cells based on CdS with or without HHST.

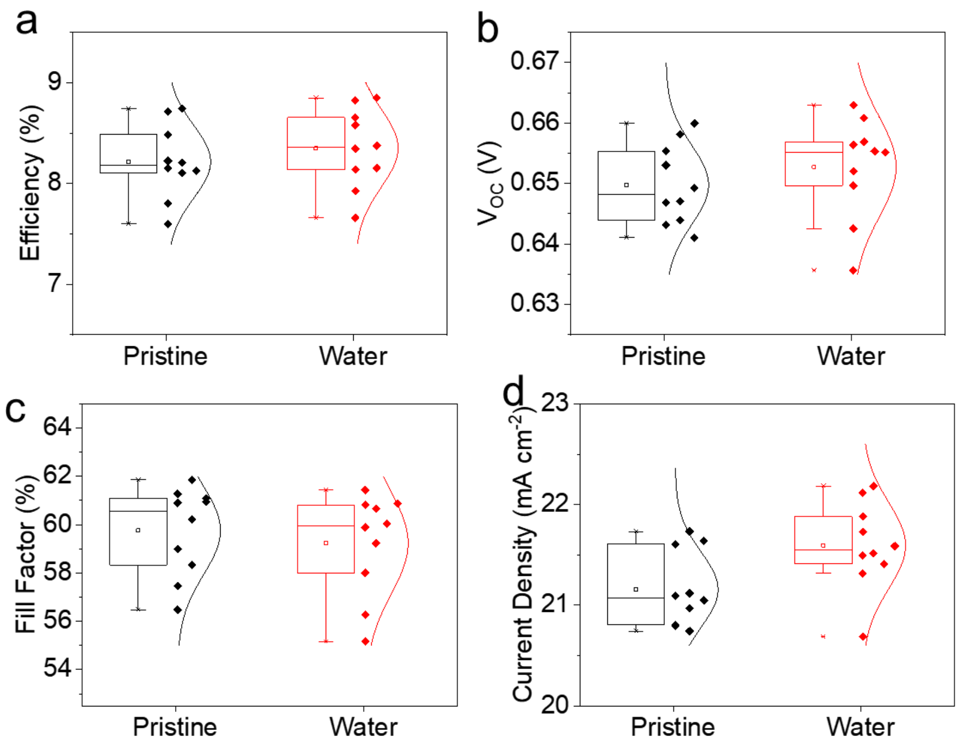

Figure S11 Statistical boxplots of $Sb_2(S,Se)_3$ solar cells' efficiency (%), Voc (V), fill factor (%), and current density (mA cm^{-2}) for pristine CdS and just treated by water.

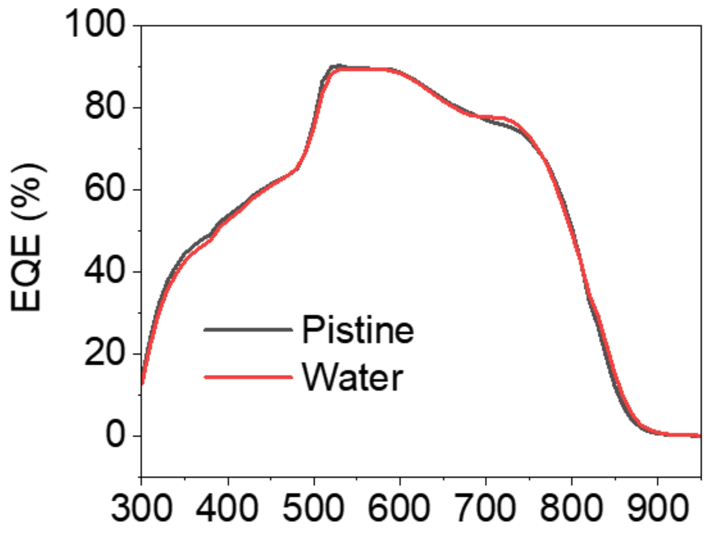

Figure S12 EQE spectrum of two types of devices, pristine CdS and just treated by water.

Table S1 Carrier lifetimes obtained from TAS decay kinetics at 632 nm curves for control and NaF-SPT $Sb_2(S,Se)_3$ films. The measured $Sb_2(S,Se)_3$ films were deposited on FTO/CdS (Control) and FTO/CdS-N_2H_4 (W-HHST).

Samples	A_1	τ_1 (ps)	A_1	τ_2 (ns)	τ_{av} (ns)
Control	0.115	1629	0.808	8687	8.50
W-HHST	0.267	59	0.772	6918	6.89
